# Ultrasonographic Assessment of Diaphragmatic Function and Its Clinical Application in the Management of Patients with Acute Respiratory Failure

**DOI:** 10.3390/diagnostics13030411

**Published:** 2023-01-23

**Authors:** Marina Saad, Stefano Pini, Fiammetta Danzo, Francesca Mandurino Mirizzi, Carmine Arena, Francesco Tursi, Dejan Radovanovic, Pierachille Santus

**Affiliations:** 1 Division of Respiratory Diseases, Ospedale Luigi Sacco, Polo Universitario, ASST Fatebenefratelli-Sacco, 20157 Milano, Italy; 2 Department of Biomedical and Clinical Sciences (DIBIC), Università degli Studi di Milano, 20157 Milano, Italy; 3 Servizio di Cardiologia, Centro Ospedaliero Militare di Milano, Esercito Italiano, 20147 Milano, Italy; 4 UOS di Pneumologia, Ospedale di Codogno, ASST Lodi, 26845 Codogno, Italy

**Keywords:** diaphragm dysfunction, ultrasound, acute respiratory failure, mechanical ventilation, muscle weakness, dyspnea

## Abstract

Acute respiratory failure (ARF) is a common life-threatening medical condition, with multiple underlying etiologies. In these cases, many factors related to systemic inflammation, prolonged use of steroids, and lung mechanical abnormalities (such as hyperinflation or increased elastic recoil due to pulmonary oedema or fibrosis) may act as synergic mechanisms leading to diaphragm dysfunction. The assessment of diaphragm function with ultrasound has been increasingly investigated in the emergency department and during hospital stay as a valuable tool for providing additional anatomical and functional information in many acute respiratory diseases. The diaphragmatic ultrasound is a noninvasive and repeatable bedside tool, has no contraindications, and allows the physician to rapidly assess the presence of diaphragmatic dysfunction; this evaluation may help in estimating the need for mechanical ventilation (and the risk of weaning failure), as well as the risk of longer hospital stay and higher mortality rate. This study presents an overview of the recent evidence regarding the evaluation of diaphragmatic function with bedside ultrasound and its clinical applications, including a discussion of real-life clinical cases.

## 1. Introduction

Acute respiratory failure (ARF) is a common life-threatening medical condition, with multiple underlying etiologies. Diaphragm muscle dysfunction is increasingly recognized as an important element that can present in patients with ARF or who are critically ill [1,2]. As of today, there are various static and dynamic imaging techniques used for the assessment of diaphragm function [3]. Static imaging techniques can be used to assess the position, shape, and dimensions of the diaphragm and include chest radiography [4], brightness mode (B-mode) ultrasound [5], computed tomography (CT) [6], and static magnetic resonance imaging (MRI) [7]. Dynamic imaging techniques are used to assess diaphragm motion in one or more directions, and include fluoroscopy [8], motion-mode (M-mode) ultrasonography [5,9], and dynamic MRI [10].

As compared with standard diagnostic approaches, chest ultrasound has been proven to improve the overall diagnostic accuracy in critical care settings, as well as in the emergency department [11]. In this context, the assessment of diaphragm function with ultrasound has been increasingly investigated in the emergency department [12,13,14] and during hospital stay [15,16] as a valuable tool for providing additional anatomical and functional information in many acute respiratory diseases [17].

The diaphragmatic ultrasound is a noninvasive and repeatable bedside tool, has no contraindications [15], and allows the physician to rapidly assess the presence of diaphragmatic dysfunction; this evaluation helps in estimating the need for mechanical ventilation (and its risk of failure), as well as the risk of longer hospital stay and higher mortality rate [13,14,15,16].

An overview of the recent evidence regarding the evaluation of diaphragmatic function with bedside ultrasound and its clinical applications is hereby presented, including a discussion of real-life clinical cases.

## 2. Diaphragm Function Assessment

The diaphragm is an ample dome-shaped muscle which separates the thoracic cavity from the abdomen. It is comprised of three different portions: the central tendon, which is a non-contractile, fibrous structure; the costal portion, which inserts to the rib cage and thoracic vertebrae; and the crural portion, which inserts to the upper three lumbar vertebrae [3]. The area in which the diaphragmatic fibers connect to the rib cage is called the zone of apposition [18].

The diaphragm is the main respiratory muscle. During inspiration, its contraction lowers and widens the inferior part of the thorax, thus lowering the intrathoracic pressure (as well as increasing the abdominal pressure) and allowing the insufflation of the lungs. During exhalation, the relaxation of the diaphragm allows the elastic recoil of the lungs to increase pleural pressure, thus removing air from the lungs [19]. In healthy individuals during resting breathing, inspiration is an active process, while exhalation is passive and does not require muscle activity [20].

Assessing the anatomy and function of the diaphragm can be an auxiliary or, in many conditions, an invaluable tool to diagnose, characterize, and monitor both acute and chronic respiratory diseases. Ultrasonography is particularly promising for this purpose, due to its availability, fast execution, repeatability, low costs, and safety; moreover, it can be easily deployed bedside, which is very helpful in the acute or critical care setting [3,18,19,20,21].

Ultrasonography of the diaphragm was first described at the end of the 1960s, but the true cornerstone of its implementation in the clinical practice was the seminal study by Wait and collaborators in 1989 [15]. Wait introduced a technique to measure the thickness of the diaphragm by applying a high-frequency ultrasound probe on the apposition zone of the diaphragmatic dome [22].

The technique to perform diaphragmatic ultrasonography has been currently revised in an Expert Consensus statement, relatively to its adoption in the Critical Care setting [23]. The diaphragm can be visualized by deploying high-frequency (10–15 MHz) probes between the seventh and the ninth intercostal space on the median anterior axillary line, transversal to the ribcage. Since the thickness of the diaphragm is variable over the surface of the muscle, a precise note regarding the position of the probe is essential to ensure the repeatability of this measure on the same patient, or to compare the values found in different patients.

**Figure 1:**
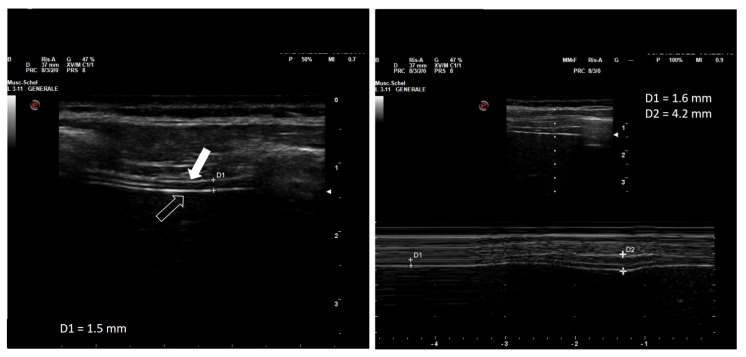
Thickness of the right hemi-diaphragm at the zone of opposition (**left** panel) and diaphragm thickening during maximal inspiration (**right** panel) in a healthy woman in her thirties while semi-recumbent. Left panel: note the two hyperechoic lines (arrows) representing the peritoneum (empty arrow) and parietal pleura (filled arrow). The measured thickness (D1) is 1.5 mm. Right panel: the same diaphragmatic region during maximal inspiration from resting volume to total lung capacity. Diaphragmatic thickening is examined in motion mode (M-Mode). During full inspiration, thickness increases from 1.6 (D1) to 4.2 mm (D2), equal to a thickening fraction of 162%. Own image.

The diaphragm can be visualized as a three-layered band, which includes (from the outer to the inner layer) the hyperechogenic diaphragmatic pleura, the relatively hypoechogenic muscle, and the hyperechogenic peritoneal pleura (Figure 1).

The distance between the inner margins of the diaphragmatic and peritoneal sheets is defined as the thickness of the diaphragm, the normal median (interquartile range) value of which is highly variable, with the majority of measurements ranging 3.3 (1.3–7.6) mm. Thickness is lower in female than in male patients, and is positively correlated to height and body weight, but independent of age [24,25]. Moreover, it should be noted that diaphragm thickness is increased in standing and sitting position compared with the recumbent position [26], and that it is greater at the lower intercostal spaces [27]. Diaphragm thickness at rest in healthy patients is highly variable, ranging from 1.2 to 11.8 mm among individuals, with group mean values of 1.6 to 3.4 mm and a lower limit of normal (LLN) in adults ranging from 0.80 to 1.60 mm [21].

Variations of the thickness of the diaphragm during the respiratory cycle are inversely proportional to variations of its transverse surface, which results from its contractile activity. Since the volume of the muscle is constant, the shortening and flattening of the diaphragm during contraction is associated by a proportional increase in its thickness (Figure 1) [22]. The percentage by which the thickness increases from the end of exhalation to the end of inspiration is called the thickening fraction, while the ratio between the end-inspiratory and the end-expiratory thickness is called the thickening ratio [28]. Similar to diaphragmatic thickness, the thickening fraction also shows wide variability in different individuals, with reference values ranging from 60% to 260% (left hemidiaphragm) and from 57 to 200% (right hemidiaphragm) while seated [28].

Assessment of the diaphragmatic thickening fraction shows good intra- and inter-observer repeatability also in mechanically ventilated patients [16,29,30].

It has been demonstrated that, in sedated and mechanically ventilated patients, passive inflation of the lungs causes an increase in the diaphragmatic thickness only when a volume of air superior to 50% of the total lung capacity is insufflated; therefore, at lower lung volumes, the thickening fraction reflects the diaphragmatic activity rather than passive alterations of the diaphragm induced by chest inflation, hence the utility of this assessment even in ventilated patients [16,27].

The thickening fraction has been widely studied as an indicator of the contractile efficiency of the diaphragm [31], and consequently as a possible predictor of the outcome of a weaning trial in mechanically ventilated patients [32,33]. A pilot study also found a correlation between the thickening fraction and the response to non-invasive support with continuous positive airway pressure (CPAP) in patients hospitalized with SARS-CoV2 pneumonia [34]. However, results from clinical study exploring the usefulness of the thickening fraction as a predictor of the outcome in patients undergoing invasive or non-invasive ventilation for acute respiratory failure are often inconclusive [35,36].

In fact, despite the fact that some studies have found a positive correlation between the thickening fraction and the pressure output of the diaphragm [37,38], the correlation between these measurements is usually weak [39,40], highlighting the need for further studies to validate the clinical applications of this technique [38].

**Figure 2:**
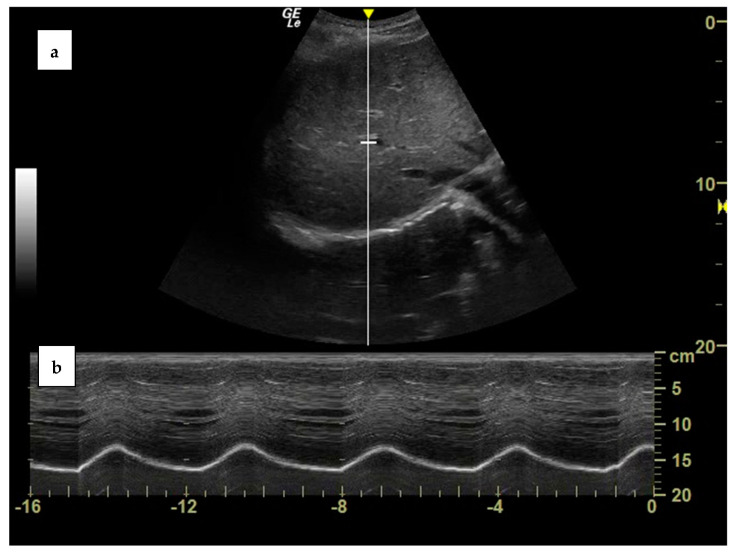
Visualization of the diaphragmatic dome from the right subcostal view in brightness (B) mode (**a**) and of the diaphragmatic excursion in motion (M) mode (**b**) in a healthy subject. Own image.

Ultrasonography also allows the measurement of the diaphragmatic cranio–caudal excursion during the respiratory cycle. This requires employing curvilinear, low-frequency probes (2–6 MHz), which can be positioned just below the right costal arch, on the right midclavicular line, and directed upwards and laterally, so that the liver acts as an acoustic window and the ultrasound beam reaches the dome of the diaphragm perpendicularly. The diaphragm will be visualized as a bright, hyperechogenic line which lays over the profile of the liver. After having obtained a good visualization of the diaphragm in 2D brightness (B) mode, ultrasonography will be switched to movement (M) mode, in order to visualize the diaphragmatic respiratory excursions over time [41] (Figure 2). The same procedure can also be executed by pointing the probe towards the left midclavicular line, thus using the spleen as an acoustic window, or on the right or left mid axillary line, even if visualization of the diaphragm is often suboptimal [9]. The maximum diaphragmatic excursion can be measured as the difference between the position of the diaphragm at functional residual capacity (FRC) and total lung capacity (TLC), while tidal excursion is the difference between the positional FRC and the end-inspiratory position during resting breathing. Ultrasonography assessment of the diaphragmatic excursion has been proven to have a good inter- and intra-observer reproducibility (Table 1) [24,26].

Diaphragmatic ultrasonography can be limited by a poor acoustic window, for example in obese patients, in the presence of marked abdominal meteorism, or in patients with massive contraction of abdominal muscles during exhalation. Moreover, complete visualization of the diaphragm over the entire respiratory cycle may be impaired in patients with ribcage anomalies. As a result, diaphragmatic excursion may be hardly measurable in up to 28% of patients [42]. Interpreting the diaphragmatic excursion is also complicated by mechanical ventilation, since it is impossible to discriminate if the lowering of the dome may be due to passive inflation of the lungs by means of the ventilator, rather than by muscular activity [37].

Due to the challenges of the ultrasound assessment of the diaphragm, the learning curve of this procedure is likely to require significant practice before being reliably implemented in the clinical setting. A randomized controlled educational study performed in eight Italian University hospitals in 2019 showed that integrating theoretical formation with practical training was able to achieve much better results in educating medical students or residents to perform diaphragmatic ultrasound [43]; the authors suggested that 25 supervised examinations could be sufficient to achieve sufficient familiarity to employ the technique in the critical care setting, similar to what has been observed regarding the training to perform lung ultrasound [44]. A study performed in 2018 involving the measurement of the diaphragmatic thickening fraction showed that intra-observer reproducibility significantly increased over the first ten patients and only slightly improved afterwards [29].

Both the thickening fraction and the excursion can be used as indexes of diaphragmatic contractility, and thus should be included in the functional evaluation of the diaphragm both in acute and chronic conditions. These parameters, and particularly the thickening fraction, have been explored as non-invasive predictors of ventilation or weaning failure in mechanically ventilated patients in the ICU setting, as discussed above [16,25,42,45,46,47,48]. There is no established consensus yet regarding the superiority of either diaphragmatic thickening fraction or excursion as a predictor of clinical outcomes in the intensive care setting, with some studies suggesting a superiority of the former [49,50] or latter [51,52,53] technique. A systematic review published in 2021 concluded that the thickening fraction had slightly lower sensitivity but higher specificity than the diaphragmatic excursion in predicting successful weaning from mechanical ventilation [54], but the analysis was potentially hindered by the significant heterogeneity between the studies. However, a more recent systematic review concluded for the superiority of the thickening fraction both in terms of sensitivity, specificity, and diagnostic Odds Ratio [33].

The gold standard procedure to assess inspiratory efforts is measuring the diaphragmatic pressure–time product (PTPdi) by means of gastric and esophageal balloons; experimental studies have demonstrated that the diaphragmatic thickening fraction has a significant, but often-underwhelming, correlation with PTPdi [37,39,55,56], while, to date, no study has found a significant correlation between diaphragmatic excursion and PTPdi [37].

## 3. Clinical Application of Diaphragm Ultrasound

### 3.1. Clinical Case One

B.M., an obese (body mass, index, BMI, 31 kg/m^2^) 75-year-old female with no smoking history, was admitted to the emergency department in October 2021 for progressive dyspnea and constrictive chest pain worsened in the last days. Her past medical history included moderate aortic–stenosis and mitral insufficiency, permanent atrial fibrillation, and arterial hypertension. Her chronic pharmacological therapy consisted of a beta-blocker, DOACs, and statin.

A doppler echocardiography revealed a worsening of her mitral insufficiency conditioning pulmonary edema. Accordingly, she was admitted to the cardiology unit, where she received a cardiopulmonary resuscitation for cardiac arrest. Subsequently, she underwent cardiac surgery that consisted of single coronary artery bypass, mitral valvuloplasty, and aortic valve replacement with a bio-prothesis.

**Figure 3:**
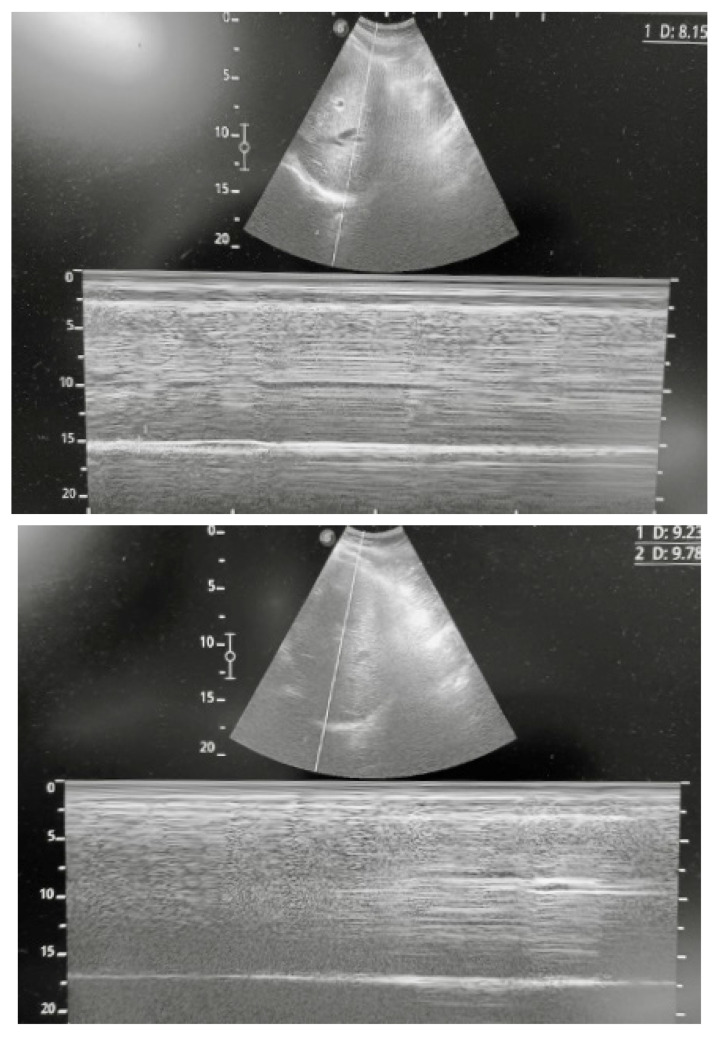
Ultrasound evaluation of B.M.’s right hemidiaphragm during resting breathing; upper image, end-exhalation, lower image, end-inhalation. B-mode and M-mode were used for the evaluation of the diaphragm excursion: it can be easily noted that there were no diaphragm movements during resting breathing.

She was then transferred to the intensive care unit (ICU), where she was mechanical ventilated. After 3 days, a first trial of extubation was performed, but reintubation was necessary after 6 h. After a week, she underwent percutaneous tracheostomy. Her clinical course in the ICU was complicated by ventilator-associated pneumonia (VAP), with isolation of P. mirabilis on broncho-alveolar lavage treated with ceftazidime, and by methicillin-resistant *S. aureus* (MRSA) septicemia, treated with daptomycin. Her weaning from tracheostomy was complicated by fever and a further increase in bronchial secretions, which occurred a week after starting antibiotic therapy. A chest HRCT was performed, revealing a mild right pleural effusion, associated with atelectasis of the lower lobe and elevation of the right hemidiaphragm. Given the recent heart surgery, which might have been complicated by phrenic nerve injury, and the presence of right hemidiaphragm elevation, diaphragm ultrasound was performed. Right hemidiaphragm dysfunction was confirmed by the absence of diaphragmatic excursion, in association with reduced muscle thickness and absence of diaphragmatic thickening (Figure 3). Phrenic nerve injury and right hemidiaphragm paresis were then confirmed by electromyography.

### 3.2. Clinical Case Two

**Figure 4:**
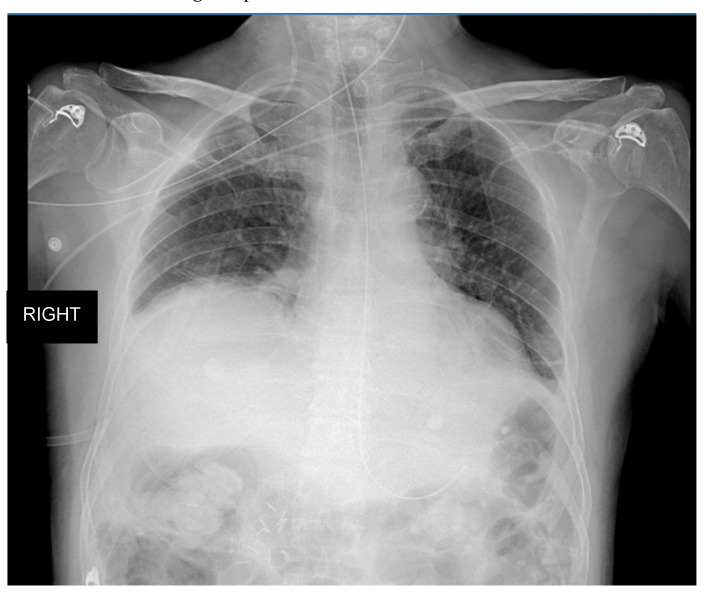
Chest X-ray performed in our unit after clinical worsening of the patient, which documented the presence of right hemidiaphragm elevation.

D.G., a 76-year-old former smoker (25 PKY), with a past medical history of arterial hypertension and a recent diagnosis of pheochromocytoma, was admitted to the intensive care unit (ICU) for acute respiratory failure which occurred after a right adrenalectomy to remove the pheochromocytoma in June 2022. Due to the high risk of cardiocirculatory arrest, he was intubated and mechanically ventilated for 3 days. After extubation, the patient was transferred to our high-dependency respiratory unit, where he was treated with non-invasive ventilation (NIV) and high-flow nasal oxygen for a relapse of respiratory distress and hypoxic respiratory failure. The chest X-ray showed the presence of bilateral inflammatory infiltrates and right hemidiaphragm elevation (Figure 4). Considering the subsequent difficult weaning from NIV, conditioned by the rapid onset of rapid shallow breathing and early respiratory fatigue, we decided to rule out the presence of diaphragmatic dysfunction. The chest ultrasound revealed the presence of a significant elevation of the right hemidiaphragm, associated with pleural effusion and partial atelectasis of the basal lung parenchyma. Although right hemidiaphragm excursion was reduced (hypomobility), the diaphragmatic thickness was within normal limits, the estimated end-expiratory thickness of the right hemidiaphragm being within 2.9 and 4.7 mm. The latter finding allowed us to exclude a diagnosis of diaphragmatic dysfunction; this assessment was later confirmed by the electromyography (EMG) of the diaphragm, that excluded a damage of the phrenic nerve. The patient was therefore treated with large spectrum antibiotic therapy, systemic steroids, and continued with NIV cycles with progressive reduction of the support pressure until weaning from the ventilatory support was achieved. A chest X-ray performed in 2015 and brought by patients’ relatives by the end of the hospital stay, showed a right hemidiaphragm elevation, confirming the pre-existence of the anatomical alteration.

### 3.3. Case Discussion

Patients admitted to the emergency department or to the intensive care unit because of acute respiratory failure have considerably high in-hospital and one-year mortality rates [57]. Diaphragm muscle dysfunction can be involved in many cases of ARF. Diaphragm dysfunction can present as weakness, paralysis, and eventration [11]; ultrasound evaluation allows the identification or exclusion of diaphragm weakness and paralysis by the presence of abnormalities of thickness, thickening, and motion, as presented in the previous clinical cases. Therefore, ultrasound assessment of the diaphragm might play a central role in the clinical and therapeutical management of the patient.

Diaphragm ultrasound has been already successfully used in patients admitted to the emergency department with ARF, and particularly in patients with acute exacerbation of chronic obstructive pulmonary disease (AECOPD) [12,13].

Marchioni and colleagues evaluated the usefulness of early and noninvasive US diaphragm evaluation in patients admitted for acute exacerbation of COPD. They reported that the presence of diaphragm dysfunction on admission, defined as a change in diaphragm thickness less than 20%, was associated with a sixfold increased risk of NIV failure within the first 48 h and a fivefold higher risk of dying during the follow up [13].

Moreover, Bobbia and colleagues proved that diaphragmatic excursion is feasible and reproducible (at both intra and inter observer level) [12]. They also reported that, in patients presenting with acute dyspnea or ARF admitted to the emergency department, an excursion higher than 2.3 cm at admission was not correlated with the necessity of NIV [12].

Diaphragm dysfunction can present also as a complication of thoracic surgery, particularly after coronary artery bypass graft (CABG) [58]. In these cases, the presence of diabetes, hypertension, and obesity are risk factors for post-operative diaphragm dysfunction [59]. The consequences of diaphragm dysfunction can be severe, resulting in an increase in mechanical ventilation duration, risk of re-intubation, as presented in our clinical case, and higher risk of mortality [60,61].

To prevent the occurrence of post-surgery diaphragmatic impairment, it has been speculated that diaphragm ultrasound might also help the assessment of pre-operative risk, in addition to the EuroSCORE II (European System for Cardiac Operative Risk Evaluation), in patients undergoing cardiac surgery [62].

More recently, Chen and colleagues analyzed the use of bedside diaphragm ultrasound in septic patients and acute respiratory failure in ICU [63]. They observed that the presence of diaphragm dysfunction, defined as reduced excursion, occurred earlier than diaphragm atrophy in septic patients. The severity of the diaphragm dysfunction was also correlated with the severity of the disease and proved to be a predictor of poor outcomes in ICU patients.

Several risk factors for diaphragmatic dysfunction during ARF have been identified (Table 2) [60,64]. Acute diaphragm injury and weakness may result from sepsis, trauma, systemic inflammation, or mechanical ventilation [65]. Demoule and colleagues identified sepsis as a major independent risk factor for diaphragm dysfunction on ICU admission [66]. Diaphragm weakness may also be a pre-existent condition and precipitate respiratory failure [47].

Formenti and colleagues demonstrated how ultrasound characteristics of the diaphragm and skeletal muscles such as rectus femoris and intercostals are strongly associated with ICU unfavorable outcomes in intubated patients with COVID-19 and ARDS [67]. More specifically, muscle echogenicity at ICU admission, quantitatively defined by a greyscale analysis, was significantly lower in survivors in respect to patients that died during the ICU stay, indicating that monitoring of diaphragm architecture in association with function can potentially become a reliable predictor of ICU survival and a possible indicator of the need for early introduction of pharmacological and non-pharmacological therapies capable of preserving muscular architecture and fitness in patients exposed to invasive mechanical ventilation. The same group has recently demonstrated that ICU-acquired weakness cannot be predicted by short-term variations that occur in diaphragmatic thickness in ventilated patients [68]. In fact, the role of diaphragmatic ultrasound to assess ICU-acquired diaphragmatic dysfunction has been lately investigated as a possible tool for predicting weaning failure in mechanically ventilated patients. The process of weaning is complex and challenging, and the occurrence of weaning failure depends upon numerous factors, among which are respiratory muscle weakness and fatigue [69]. Respiratory muscle weakness, and in particular diaphragmatic dysfunction, can develop secondary to pre-existing conditions (such as neuromuscular disorders, malnutrition, static and dynamic hyperinflation, endocrine disturbances) and to ventilator-associated muscle dysfunction, sepsis-associated neuro-myopathy, ICU-acquired paresis, and disturbances related to disease and treatment such as severity of respiratory failure and medications [69], and can derive from an increased resistive and elastic load, or from a deficiency of the contractile properties of the muscle. Therefore, ultrasound-guided evaluation of diaphragmatic performance has been proposed as a tool to guide the readiness for a weaning trial and predict its outcome. Al Tayar et al. showed that, compared with the electrical activity of the diaphragm, diaphragmatic thickening fraction performed better as a predictor of weaning failure [70]. Two-dimensional speckle tracking to detect diaphragm longitudinal strain has been studied as an index of diaphragmatic dysfunction capable of predicting weaning outcome. Despite acceptable sensitivity and fair specificity, longitudinal strain did not perform better that the rapid shallow breathing index in predicting liberation from mechanical ventilation [71]. Finally, Palkar and colleagues have proposed the excursion–time index of the diaphragm to estimate the diaphragmatic respiratory work and, consequently, its potential role as a predictor of weaning failure. The authors suggested that an increase in or the maintenance of the excursion–time index (the product of diaphragmatic excursion and inspiratory time) following the suspension of assisted ventilation during a spontaneous breathing trial can be a reliable predictor of weaning outcome [48]. Despite its promise, to date these indexes have not been validated with the gold standard measurements of diaphragmatic performance (e.g., pressure time product of the diaphragm), and still cannot properly reflect the contribution of the diaphragm or the involvement of accessory respiratory muscles during the different phases of a weaning process.

## 4. Conclusions

The diaphragm is the main muscle involved in ventilation and whole-body homeostasis. In patients with respiratory impairment or acute respiratory distress due to different etiologies (AECOPD, sepsis, thoracic surgery, pneumoniae, etc.), many factors related to systemic inflammation, prolonged use of steroids, and lung mechanical abnormalities (such as hyperinflation or increased elastic recoil due to pulmonary oedema or fibrosis) may act as synergic mechanisms leading to diaphragm dysfunction [72]. This explains the heterogeneity of clinical conditions that can be associated with diaphragm abnormalities, as previously described. On the other hand, diaphragmatic impairment is a negative prognostic factor in both chronic and acute respiratory diseases, possibly increasing mortality, length of hospitalization, and risk of treatment failure [73,74]. Since diaphragm ultrasound has proved to be a feasible diagnostic tool in subjects with acute respiratory conditions, including ARF, acute dyspnea, sepsis, or prolonged use of steroids [63], it can be easily understood how useful it can be for the physician to evaluate the diaphragm with ultrasonography in an emergency setting, ICU, or during hospital stay.

In conclusion, assessment of diaphragmatic anatomy and function by bedside ultrasonography should be encouraged as an integral part of clinical practice in respiratory care.

## Figures and Tables

**Table 1:** Normal reference values of diaphragm ultrasound measurements expressed as medium (5–95° percentile). Adapted from: Goligher EC. Diaphragm Ultrasound. In: Magder S, Malhotra A, Hibbert KA, Hardin CC (Editors). Cardiopulmonary Monitoring. Basic Physiology, Tools, and Bedside Management for the Critically Ill. Springer Nature Switzerland AG 2021.

Measurement	Normal Range
End-expiratory thickness of the right hemidiaphragm	3.3 mm (0.17 mm–5.3 mm)
Thickening fraction during resting breathing	20% (5–50%)
Maximum diaphragmatic thickening fraction	80% (20–180%)
Diaphragmatic excursion during resting breathing	1.7 cm (1.0–2.5 cm)
Diaphragmatic excursion during a maneuver of inspiratory capacity	6.5 cm (3.6–9.2 cm)

**Table 2:** Risk factors for diaphragmatic dysfunction.

Risk Factors for Diaphragmatic Dysfunction
**Acquired Conditions**
Sepsis Prolonged mechanical ventilation ICU acquired paresis/neuromiopathy Trauma Medications
**Pre-Existent Conditions**
Neuromuscular disorders COPD Hyperinflation Malnutrition

## Data Availability

Not applicable.
